# Development of evidence-based clinical algorithms for prescription of exercise-based cardiac rehabilitation

**DOI:** 10.1007/s12471-015-0761-y

**Published:** 2015-10-19

**Authors:** R.J. Achttien, T. Vromen, J.B. Staal, N. Peek, R.F. Spee, V.M. Niemeijer, H.M. Kemps, on behalf of the multidisciplinary expert panel

**Affiliations:** 1 Radboud Institute for Health Sciences, Radboud University Medical Center, IQ healthcare, Geert Grooteplein 21, 6500 HB Nijmegen, The Netherlands; 2 Department of Medical Informatics, University of Amsterdam, Amsterdam, The Netherlands; 3 Institute Health Studies, HAN University of Applied Sciences, Nijmegen, The Netherlands; 4 Health e-Research Centre, University of Manchester, Manchester, UK; 5 Department of Cardiology, Máxima Medical Centre, Veldhoven, The Netherlands; 6 Department of Sports Medicine, Elkerliek Hospital, Helmond, The Netherlands

**Keywords:** Clinical algorithms, Exercise-based, Prescription, Cardiac rehabilitation, Coronary artery disease, Chronic heart failure

## Abstract

**Background:**

Guideline adherence with respect to exercise-based cardiac rehabilitation (CR) is hampered by a large variety of complex guidelines and position statements, and the fact that these documents are not specifically designed for healthcare professionals prescribing exercise-based CR programs. This study aimed to develop clinical algorithms that can be used in clinical practice for prescription and evaluation of exercise-based CR in patients with coronary artery disease (CAD) and chronic heart failure (CHF).

**Methods:**

The clinical algorithms were developed using a systematic approach containing four steps. First, all recent Dutch and European cardiac rehabilitation guidelines and position statements were reviewed and prioritised. Second, training goals requiring a differentiated training approach were selected. Third, documents were reviewed on variables to set training intensity, modalities, volume and intensity and evaluation instruments. Finally, the algorithms were constructed.

**Results:**

Three Dutch guidelines and three European position statements were reviewed. Based on these documents, five training goals were selected and subsequently five algorithms for CAD patients and five for CHF patients were developed.

**Conclusions:**

This study presents evidence-based clinical algorithms for exercise-based CR in patients with CAD and CHF according to their training goals. These algorithms may serve to improve guideline adherence and the effectiveness of exercise-based CR.

## Introduction

Multidisciplinary cardiac rehabilitation (CR) reduces mortality and morbidity and prevents recurrence of cardiac events and hospitalisation in patients with coronary artery disease (CAD) and chronic heart failure (CHF) [1, 2]. Exercise-based CR constitutes an important part of outpatient multidisciplinary CR and has been shown to improve exercise capacity and quality of life [3–5].

It is widely recognised that the effectiveness of exercise-based CR highly depends on training methods, and that results can be improved when the contents of training programs are tailored to the patients’ personal goals and baseline exercise capacity [6]. In addition, exercise-based CR needs to be evidence-based and disease-specific with respect to the contents and safety criteria. Currently, various comprehensive exercise-based CR guidelines and position papers exist [7–14]. However, the training principles described in these documents are not well implemented in daily practice. A recent survey among 45 Dutch CR centres showed that considerable variation exists in methods for determination of exercise capacity, training intensity and volume [15]. In addition, recommended assessment methods (e.g. symptom-limited exercise testing) were often not used, nor standardised. These results are in line with studies in other countries, also showing poor implementation of exercise-based CR guidelines [16–18].

A strategy that may be used to improve implementation of multiple complex guidelines is the development of clinical algorithms [19]. Clinical algorithms are flowcharts highlighting the information that needs to be gathered for advising on optimal treatment for a given individual, thereby aiming to reduce practice variation and increase guideline adherence. The main purpose of the present study was to develop evidence-based clinical algorithms that can serve as best practice standards for prescription and evaluation of exercise-based CR in patients with CAD and CHF.

## Methods

The clinical algorithms were composed by a multidisciplinary expert panel consisting of cardiologists, physiotherapists, sports physicians, occupational physicians, a rehabilitation physician, a human movement scientist and a health informatician. A psychologist was included on consultation basis. All experts were mandated by their national societies. The algorithms were developed to serve as an implementation tool of guidelines for all patients with an indication for exercise-based CR according to the current guidelines. This document addresses all diagnoses or indications for CR for which there are clear recommendations in the guidelines and position statements. As such, the algorithms were developed for CAD patients who have an absolute indication for CR [20], namely:

- Acute coronary syndrome, including ST and non-ST-elevation myocardial infarction and unstable angina pectoris;
- Stable angina pectoris;
- Acute or elective percutaneous coronary intervention;
- Coronary artery bypass grafting and/or valve surgery;
- Chronic heart failure (persistent reduction of left ventricular ejection fraction < 40 %) [20].

The clinical algorithms were developed stepwise:

1. Selection and prioritisation of guidelines and position statements;
2. Selection of training goals;
3. Data extraction and synthesis;
4. Construction of algorithms.

## Selection and prioritisation of guidelines and position statements

All Dutch CR guidelines and recently published position statements from the European Society of Cardiology (ESC) were assessed for their relevance. For selection and prioritisation, the following order was applied:

- First national guidelines were consulted, then ESC position statements.
- General CR guidelines were consulted prior to disease-specific guidelines.

## Selection of training goals

According to the Dutch algorithm for patient needs in CR 2012, 19 exercise-based CR goals can be discerned [21]. The members of the expert panel were instructed to cluster goals requiring a similar training approach according to the selected exercise-based CR guidelines and position statements.

## Data extraction and synthesis

A systematic search was conducted in each guideline and position statement by three researchers independently (HK, TV and RS), assessing the following items for each of the selected training goals and diagnosis group:

- Variables to set training modalities;
- Training volume and intensity;
- Contents of training programs;
- Evaluation instruments.

## Construction of clinical algorithms

Based on the selected data, clinical algorithms were constructed for each combination of diagnosis group and training goal by three panel members. The algorithms were discussed with the other panel members in several meetings and adjusted until consensus was reached. When insufficient information could be retrieved from the available literature, panel members were instructed to use expert opinion to complete the algorithms.

## Results

### Selection and prioritisation of guidelines and position statements

Available Dutch guidelines and ESC position statements on exercise-based CR were reviewed. Table 1 presents the result of the selection procedure including the prioritisation order.

**Table 1:** Guideline and position statement selection and prioritisation

1. Dutch multidisciplinary guideline for cardiac rehabilitation. Netherlands Society of Cardiology (NVVC). 2011 [20]
2. Dutch algorithm for patients needs in cardiac rehabilitation. Netherlands Society of Cardiology (NVVC). 2012 [21]
3. Dutch guidelines for exercise-based cardiac rehabilitation in coronary artery disease and chronic heart failure. Royal Dutch Society for Physiotherapy (KNGF). 2011 [7, 8]
4. Dutch national guideline for occupational medicine and labor physicians dealing with employees with coronary artery disease. Netherlands Society of Occupational Medicine (NVAB). 2006 [31]
5. Secondary prevention through cardiac rehabilitation: from knowledge to implementation. A position paper from the Cardiac Rehabilitation Section of the European Association of Cardiovascular Prevention and Rehabilitation (EACPR). 2010 [12]
6. Aerobic exercise intensity assessment and prescription in cardiac rehabilitation: a joint position statement of the EACPR, the American Association of Cardiovascular and Pulmonary Rehabilitation (AACPR) and the Canadian Association of Cardiac Rehabilitation (CACR). 2013 [11]
7. Exercise training in heart failure: from theory to practice. A consensus document of the Heart Failure Association (HFA) and the European Association for Cardiovascular Prevention and Rehabilitation (EACPR). 2011 [13]

## Selection of training goals

Eighteen of the 19 CR goals from the Dutch algorithm for patient needs in CR 2012 were clustered into five specific exercise-based CR goals that require a differentiated training approach (Table 2; [21]).

**Table 2:** Rehabilitation goal clustering

Original goals from needs assessment	Cluster
Overcoming anxiety for exercise	Reducing exercise-related anxiety
Regaining emotional balance	
Optimising exercise capacity	Optimising exercise capacity
Exploring physical limits	Exploring physical limits and coping with physical limitations
Coping with physical limitations	
Functionally managing the heart disease	
Optimal resumption of leisure activities	Developing (and maintaining) a physically active lifestyle and optimising cardiovascular risk factors
Familiarity with the nature of the disease and risk factors	
Quit smoking	
Developing and maintaining and active lifestyle	
Developing a healthy diet	
Optimising weight	
Optimising blood pressure	
Optimising diabetes management	
Optimising lipid profile	
Regaining emotional balance within relationship, family and/or social environment and work	Optimal work resumption
Optimal resumption of role within relationship, family and/or social environment and work	
Regaining emotional balance through caregiver and preventing negative effects on patients health	

## Data extraction and synthesis

According to the selected documents, CAD does not require major differences in training approach, therefore the clinical algorithms for these diagnosis groups were combined. Specific recommendations for subgroups of patients, for instance patients after cardiac surgery and patients with an implantable cardioverter defibrillator (ICD), are incorporated throughout this document.

## Construction of clinical algorithms

For all five exercise-based CR goals one clinical algorithm for both CAD and CHF patients was developed, resulting in a total of 10 algorithms. All algorithms result in a recommendation for several training modalities. Table 3 and 4 show the training recommendations for each modality, for CAD and CHF patients respectively. Training prescription for CAD patients requires assessment of exercise capacity by a symptom-limited exercise test. Exercise intensity of aerobic training in these patients should be expressed either as a percentage of heart rate reserve, peak oxygen uptake (pVO2) or, if maximal exercise cannot be performed, on the Borg rating scale of perceived exertion [22]. In CHF patients, symptom-limited exercise testing should be combined with gas exchange analysis, enabling assessment of peak oxygen uptake. If facilities are lacking, it is recommended to use a combination of a symptom-limited exercise test without gas analysis with a 6 min walk test (6MWT) [20, 23].

**Table 3:** Training recommendations for patients with stable angina pectoris, acute coronary syndrome and CABG/valve surgery

Training goal	Training modalities	Timing and frequency	Intensity and session duration	Evaluation instruments
Reducing exercise-related anxiety	Aerobic training (CT or HIT)	Week 0–4 CT or HIT: 2–3/week	CT: 50–80 % pVO2/HRR, 20–60 min	Cardiac Anxiety Questionnaire [24] at baseline, 4 weeks and 8 weeks
	Relaxation program	Week 4–8 CT at home: 2–3/week	HIT: 80–90 % pVO2/HRR, active recovery 40–50 % of pVO2/ HRR, interval 4 × 4 min, active recovery 3 × 3 min^a^	
	Education	Week 0–8 RP: 2–8 sessions	CT at home: 45–59 % pVO2/HRR or RPE-scale 11–13, 45–60 min	
Optimising exercise capacity	Aerobic training (CT or HIT)	Week 0–12: CT or HIT: 3–5/week	CT: 50–80 % pVO2/HRR, 20–60 min	Symptom-limited exercise test at baseline and final evaluation
	Resistance training	Week 0–12: RT: 2–3/week	HIT: 80–90 % pVO2/HRR, active recovery 40–50 % of pVO2/HRR, interval 4 × 4 min, active recovery 3 × 3 min^a^	6MWT [26] or SWT [25] for interim evaluation
	Functional training	Week 0–4: FT: 2–3/week	RT: 30–80 % 1RM, 8–10 exercises using large muscle groups, 2–3 sets of 10–15 repetitions, 1–2 min rest (post-CABG/valve surgery: start after 6–8 weeks)	1-RM-testing at baseline, after 2 weeks and from there on every 4 weeks [27]
	Relaxation program	Week 0–12: RP: 2–8 sessions		
	Education			
Coping with physical limitations	Aerobic training (CT or HIT)	Week 0–4 CT or HIT: 3–5/week	CT: 50–80 % pVO2/HRR, 20–60 min	PSC [28] at baseline and final evaluation
	Functional training	Week 4–8: CT at home: 2–3/week	HIT: 80–90 % pVO2/HRR, active recovery 40–50 % of pVO2/HRR, interval 4 × 4 min, active recovery 3 × 3 min^a^	
	Relaxation program	Week 0–4: FT: 2–3/week	CT at home: 45–60 % HRR or RPE scale 11–13, 45–60 min	
	Education	Week 0–8: RP: 2–8 sessions		
Developing a physically active lifestyle	Aerobic training (CT, at home))	Week 0–4 CT: 2–3/week	CT: 50–80 % pVO2/HRR, 20–60 min	Dutch Standard Healthy Movement [29], the International Physical Activity Questionnaire (IPAQ) [30] or PAEE assessment at baseline and final evaluation
	Functional training	Week 4–12 CT at home: 5–7/week	CT at home 45–59 % of pVO2/ HRR or RPE scale 11–13, 45–60 min	
	Relaxation program	Week 0–4 FT: 2–3/week		
	Education	Week 0–12 RP: 2–8 sessions		
Work resumption	Aerobic training (CT or HIT)	Week 0–12: CT/HIT: 3–5/week	CT: 50–80 % pVO2/HRR, 20–60 min	Symptom-limited exercise test at baseline and final evaluation
	Resistance training^a^	Week 0–12: RT: 2–3/week	HIT: 80–90 % pVO2/HRR, active recovery 40–50 % of pVO2/HRR, interval 4 × 4 min, active recovery 3 × 3 min^a^	6MWT [26] or SWT [25] for interim evaluation
	Functional training	Week 0–4: FT: 2–3/week	RT: work specific	1-RM-testing at baseline, after 2 weeks and from there on every 4 weeks [27]
	Relaxation program	Week 0–12: RP 2–8 sessions		
	Education			

*CT* continuous training, *HIT* high-intensity interval training, *RP* relaxation program, *RT* resistance training, *FT* functional training, *pVO2* peak oxygen uptake, *HRR* heart rate reserve, *1RM* 1 repetition maximum, *MVC* maximum voluntary contraction, *RPE* Borg rating scale of perceived exertion 6–20, *PSC* patient-specific complaints questionnaire, *PAEE* physical activity energy expenditure, 6MWT six-minute walk test, *SWT* shuttle walk test.

^a^HIT is discouraged in patients with an ICD.

**Table 4:** Training recommendations for patients with chronic heart failure (NYHA class II-III)

Training goal	Training modalities	Timing and frequency	Intensity and session duration	Evaluation instruments
Reducing exercise-related anxiety	Aerobic training (CT, HIT or LIT)	Week 0–4: CT, HIT or LIT 2–3/week	CT: 50–80 % pVO2/HRR, 20–60 min	Cardiac Anxiety Questionnaire [24] at baseline, 4 weeks and 8 weeks
	Relaxation program	Week 4–8: CT at home: 2–3/week	HIT: 80–90 % pVO2/HRR, active recovery 40–50 % of pVO2/ HRR, interval 4 × 4 min, active recovery 3 × 3 min^a^	
	Education	Week 0–8: RP: 2–8 sessions	LIT: 50 % maximal workload, 10–12 intervals 30 s, recovery 60 s	
			CT at home: 45–60 % pVO2/HRR or RPE -scale 11–13, 45–60 min	
Optimising exercise capacity	Aerobic training (CT, HIT or LIT)	Week 0–12 CT, HIT or LIT: 3–5/week	CT: 50–80 % pVO2/HRR, 20–60 min	Symptom-limited exercise test at baseline and final evaluation
	Resistance training	Week 0–12 RT: 2–3/week	HIT: 80–90 % pVO2/HRR, active recovery 40–50 % of pVO2/ HRR, interval 4 × 4 min, active recovery 3 × 3 min^a^	SWT [25] for interim evaluation
	Functional training	Week 0–4 FT: 2–3/week	LIT: 50 % maximal workload, 10–12 intervals 30 s, recovery 60 s	1-RM-testing [27] at baseline, after 2 weeks and from there on every 4 weeks
	Inspiratory muscle training	Week 0–12 IMT: 3–4/week	RT: 30–65 % 1RM, 8–10 exercises using large muscle groups, 2–3 sets of 10–15 repetitions, 1–2 min rest (post- CABG/valve surgery: start after 6–8 weeks)	
	Relaxation program	Week 0–12 RP: 2–8 session	IMT: inspiratory muscle training at 20–40 % of PiMax, 2 × 15 min/day	
	Education			
Coping with physical limitations	Aerobic training (CT, HIT or LIT)	Week 0–4 CT, HIT or LIT: 3–5/week	CT: 50–80 % pVO2/HRR, 20–60 min	PSC [28] at baseline and final evaluation
	Functional training	Week 4–8 CT at home: 2–3/week	HIT: 80–90 % pVO2/HRR, active recovery 40–50 % of pVO2/ HRR, interval 4 × 4 min, active recovery 3 × 3 min^a^	
	Relaxation program	Week 0–4 FT: 2–3/week	LIT: 50 % maximal workload, 10–12 intervals 30 s, recovery 60 s	
	Education	Week 0–8 RP: 2–8 sessions	CT at home: 45–60 % HRR or RPE scale 11–13, 45–60 min	
Developing a physically active lifestyle	Aerobic training (CT)	Week 0–4 CT 2–3/week	CT: 50–80 % pVO2/HRR, 20–60 min	Dutch Standard Healthy Movement [29], the International Physical Activity Questionnaire (IPAQ) [30] or PAEE assessment at baseline and final evaluation
	Functional training	Week 4–12 CT at home: 5–7/week	CT at home 45–60 % of pVO2/HRR or RPE scale 11–13, 45–60 min	
	Relaxation program	Week 0–4 FT 2–3/week		
	Education	Week 0–12 RP 2–8 sessions		
Work resumption	Aerobic training (CT, HIT or LIT)	Week 0–12 CT, HIT or LIT: 3–5/week	CT: 50–80 % pVO2/HRR, 20–60 min	Symptom-limited exercise test at baseline and final evaluation
	Resistance training	Week 0–12 RT: 2–3/week	HIT: 80–90 % pVO2/HRR, active recovery 40–50 % of pVO2/ HRR, interval 4 × 4 min, active recovery 3 × 3 min^a^	6MWT(26) or SWT(25) for interim evaluation
	Functional training	Week 0–4 FT: 2–3/week	LIT: 50 % maximal workload, 10–12 intervals 30 s, recovery 60 s	1-RM-testing(27) at baseline, after 2 weeks and from there on every 4 weeks
	Relaxation program	Week 0–12 IMT: 3–4/week	RT: work specific	
	Education	Week 0–12 RP: 2–8 sessions	IMT: 3–4/week (if PiMax < 70 % of predicted)	

*CT* continuous training, *HIT* high-intensity interval training, *LIT* low-intensity interval training, *IMT* inspiratory muscle training, *RP* relaxation program, *RT* resistance training, *FT* functional training, *pVO2* peak pulmonary oxygen consumption, *HRR* heart rate reserve, *1RM* 1 repetition maximum, *MVC* maximum voluntary contraction, *RPE* rate perceived exertion measured by the BORG scale (6–20), *Pimax* maximal static inspiratory mouth pressure, maximum inspiratory muscle strength, *PSC* patient-specific complaints questionnaire, *PAEE* physical activity energy expenditure, *6MWT* six-minute walk test, *SWT* shuttle walk test.

^a^HIT is discouraged in patients with an ICD.

### Reducing exercise-related anxiety

These clinical algorithms are based on the Dutch multidisciplinary guideline for CR, the Dutch algorithm for patient needs in CR and the Dutch guidelines for exercise-based CR in CAD and CHF patients [7, 8, 20, 21]. Expert opinion was used for the choice of the evaluation instrument and for the advice of a period of home-based training. These algorithms consist of two phases and three different training modalities, namely aerobic training, education and a relaxation program. During the first phase, aerobic training sessions are supervised by a physiotherapist and consist of high-intensity interval training or continuous training with gradually increasing exercise intensity (Table 3). In addition, patients receive education on how to cope with anxiety for physical exertion and insight into mechanisms causing anxiety. Also, feedback and advice is given on their daily activity pattern by relating activities from a metabolic equivalent (MET) table to their measured exercise capacity. During the second phase, patients are instructed to perform tailored aerobic training sessions in their home environment, aiming at development of self-management skills. Throughout both phases, patients participate in a relaxation program, consisting of biofeedback and breathing regulation exercises [24]. Exercise-related anxiety should be evaluated at baseline, after 4 weeks and after completion of the program, preferably by the Cardiac Anxiety Questionnaire [25]. If no improvement is observed after the initial 4-week period, patients should be referred to a psychologist. When comparing the algorithms for CHF and CAD patients, two differences can be noticed. First, in CHF patients the shuttle walk test is recommended to monitor training progression during the program [26]. Second, differences exist in the application and intensity of the aerobic training sessions (Table 3, 4). Furthermore, because there are no recommendations for patients with an ICD with respect to high-intensity interval training in the current guidelines, this training modality is in general not recommended in ICD patients. For continuous training it is recommended to perform exercise at an intensity corresponding to a heart rate of at least 20 beats/min below the ICD intervention zone [13].

### Optimising exercise capacity

**Fig 1:**
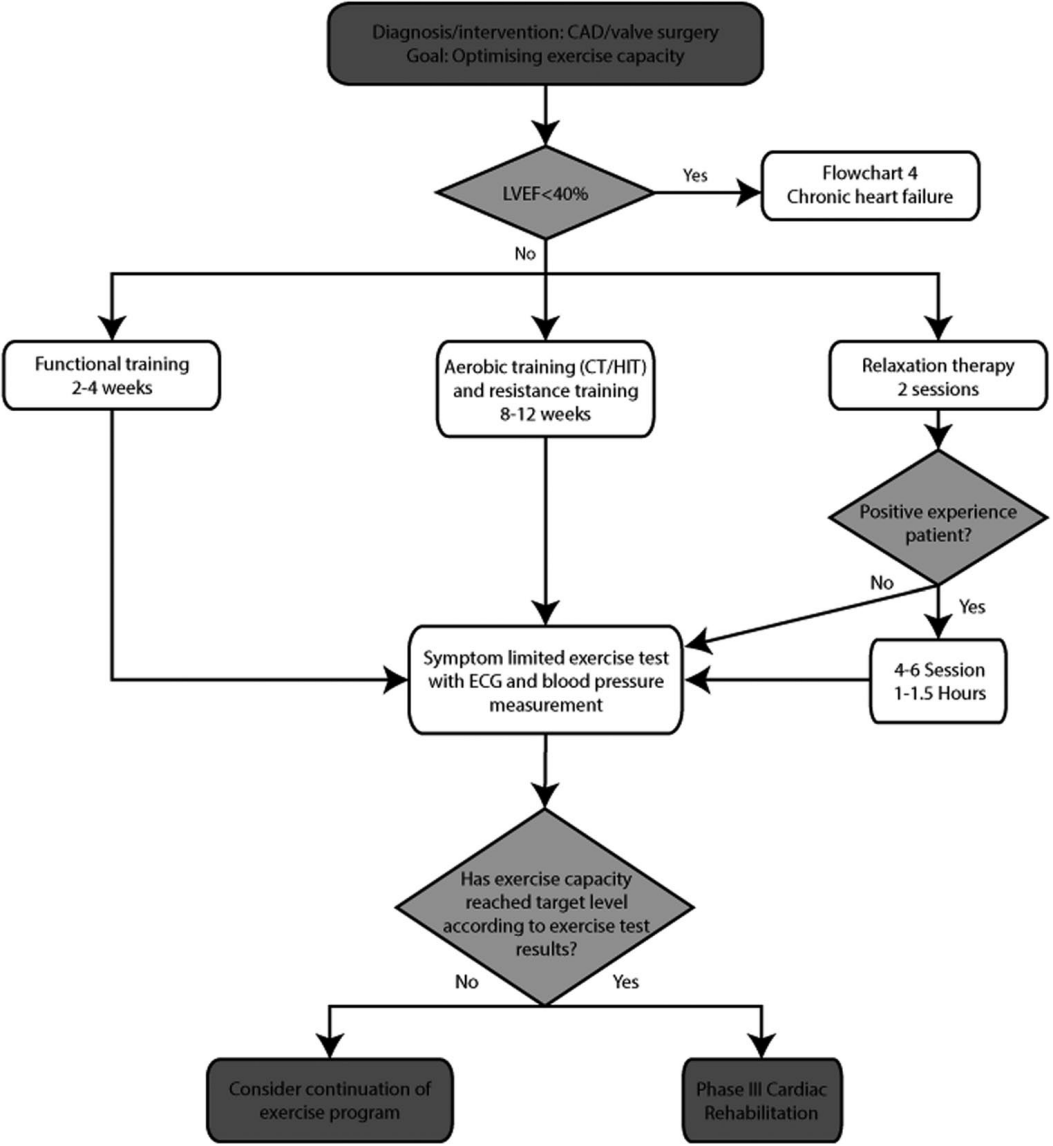
Algorithm ‘optimising exercise capacity’ for CAD patients. *CAD* coronary artery disease, *CT* continuous training, *HIT* high-intensity interval training, *LVEF* left ventricular ejection fraction

**Fig. 2:**
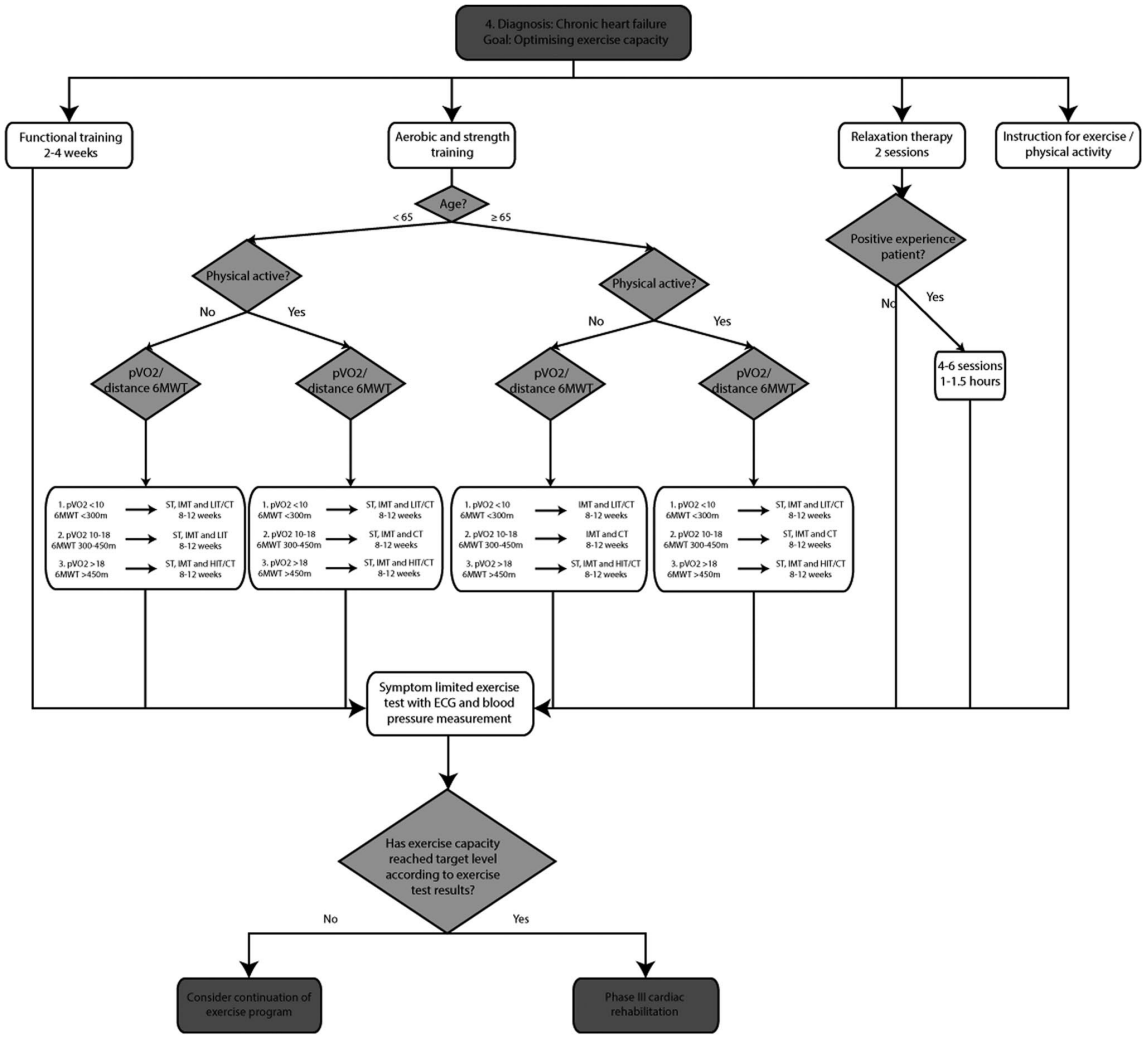
Algorithm ‘optimising exercise capacity’ for CHF patients. *LVEF* left ventricle ejection fraction, *pVO2* peak oxygen uptake (ml/min/kg), *6MWT* six-minute walk test, *CT* continuous training, *HIT* high-intensity interval training, *LIT* low-intensity interval training, *IMT* inspiratory muscle training, *RT* resistance training

These algorithms are based on the Dutch multidisciplinary guideline for CR, the Dutch algorithm for patient needs in CR, the Dutch guidelines for exercise-based CR in CAD and CHF patients and three European Association for Cardiovascular Prevention and Rehabilitation (EACPR) position statements [7, 8, 11–13, 20, 21]. Figure 1 and 2 represents the algorithm for "optimising exercise capacity" in CAD and CHF. The algorithms consist of supervised aerobic exercise training (high-intensity interval training or continuous training), resistance training, relaxation therapy and functional training. Functional training consists of specific exercises representative of daily life activities. Resistance training involves training of large muscle groups, using 2–3 sets of 10–15 repetitions separated by 1–2 min resting periods. In CAD patients, intensity should be commenced at 30–40 % of the one repetition maximum (1-RM), with a gradual increase until 50–80 % in the following 10 weeks. Resistance training is not advised during the first 6–8 weeks after cardiac surgery. In CHF patients, resistance training should commence at 30 % of 1-RM during the first 2 weeks with a gradual increase to 40–65 % of 1-RM [27]. In CHF patients, furthermore, high-intensity interval training is only recommended as an alternative for continuous training if pVO2 exceeds 18 ml/min/kg, while low-intensity interval training (Table 4) may be an alternative for continuous training in patients with a pVO2 below 10 ml/min/kg (or 6MWT distance < 300 m) [13]. Inspiratory muscle training is indicated as an adjunct to aerobic training and resistance training in CHF patients with a maximal static inspiratory mouth pressure (Pimax) below 70 % of predicted or a ventilatory impairment.

### Exploring physical limits and coping with limitations

These clinical algorithms are based on the Dutch multidisciplinary guideline for CR, the Dutch algorithm for patient needs in CR, the Dutch guidelines for exercise-based CR in CAD and CHF patients and an EACPR position statement [7, 8, 11, 20, 21]. Expert opinion was used for the advice of a period of home-based training. This algorithm is made up of two phases. The first phase consists of supervised aerobic training sessions including continuous training or high-intensity interval training supported by functional training, including practising functional skills related to problematic activities as identified by the Patient Specific Complaints questionnaire [28]. Education and advice on how to cope with physical limitations are also provided, by relating patients’ actual exercise capacity to habitual and leisure time/ sports activities, using a MET list. Home-based aerobic training is recommended in the second phase. Throughout both phases, patients participate in a relaxation program. Training volume and intensity for coping with physical limitations are assessed in the same way as for reducing exercise-related anxiety. The Patient Specific Complaints questionnaire is used to assess and grade coping behaviour with respect to problematic activities at the start and the end of the program [28]. If no improvement is observed, referral to a psychologist should be considered. For CHF, the strategy is the same, except for the application and intensity of the aerobic training sessions (Table 3, 4).

### Developing a physically active lifestyle and optimising risk factors

These algorithms are based on the Dutch multidisciplinary guideline for CR, the Dutch algorithm for patient needs in CR, the Dutch guidelines for exercise-based CR in CAD and CHF patients and two EACPR position statements [7, 8, 11, 13, 20, 21]. Expert opinion was used for the advice of a period of home-based training and the evaluation instruments. The algorithms comprise aerobic training (continuous training), functional training, education and relaxation therapy and consist of two phases. Education is focused on the development of self-efficacy and self-management skills. MET tables are used for providing patients insight into their physical activity behaviour and possibilities for improvement. During the first phase, aerobic training volume and intensity are determined in the same way as for reducing exercise-related anxiety, both for CAD and CHF patients. During home-based training patients are instructed to perform continuous training at a moderate intensity for at least 45 min for 5–7 days per week. Physical activity behaviour is evaluated by the Dutch Standard Healthy Movement Questionnaire, or the International Physical Activity Questionnaire [29, 30]. Alternatively, physical activity energy expenditure may be evaluated by an accelerometer and/or heart rate monitor.

### Optimal work resumption

These algorithms are based on the Dutch national guideline for industrial and occupational medicine, the Dutch multidisciplinary guideline for CR, the Dutch algorithm for patient needs in CR and the Dutch guidelines for exercise-based CR in CAD and CHF patients [7, 8, 20, 21]. To determine the contents of the program the average static and dynamic workload of working activities should be related to the patients’ exercise capacity. If the static workload is, on average, below 15 % of the patients’ maximal voluntary contraction and the average dynamic workload of working activities exceeds 40 % of maximal exercise capacity, the program consists mainly of aerobic training. If the average static workload exceeds 15 % of the maximal voluntary contraction, work-specific resistance training should be added. For CHF patients, the application of high-intensity interval training and the intensity of resistance training are based on the same principles as for optimising exercise capacity. All patients should furthermore be referred for relaxation therapy, functional training and receive education. Education is aimed at providing insight into the physical demands of working activities in relation to the actual exercise capacity and advice on coping with physical constraints. After completion of the program, static and dynamic workloads are reassessed. If patients are not able to resume working activities (static workload > 15 % and/or dynamic workload > 40 %) they should be sent to the company medical officer regarding possible adaption of their work situation.

## Discussion

This study is the first to present evidence-based clinical algorithms for exercise-based CR. These algorithms follow a systematic approach leading to a personalised exercise-based CR program for CAD and CHF patients by taking into account the referral diagnosis, rehabilitation goals and individual physical fitness levels. By defining evaluation instruments for each specific exercise-based CR goal, the algorithms also provide the opportunity to assess the progress towards exercise-based CR goals.

In a recent survey study in the Netherlands, it was shown that exercise-based CR guidelines were poorly implemented in daily practice [15]. This lack of guideline adherence may have various causes. Barriers to guideline compliance are commonly divided into internal and external barriers [32–34]. Internal barriers include the professional’s awareness, familiarity and attitude towards guidelines. It is known that 10 % of healthcare professionals are not aware of the existence of guidelines, with even lower familiarity with these guidelines [32]. External barriers are related to the complexity of guidelines themselves, organisational constraints (e.g. lack of facilities and time), and other environmental factors that are not directly related to the functioning of professionals [32, 35]. In addition, patient-related factors such as individual preferences and scheduling problems are mentioned as barriers to following guidelines [32, 36].

As awareness and familiarity with guidelines often constitute important barriers for guideline implementation, the existence of numerous comprehensive guidelines could hamper its implementation [7, 8, 11–13, 20, 21]. Therefore, combining and translating guidelines into clinical algorithms might improve implementation of exercise-based CR guidelines. Also, better tailoring of guidelines may reduce external barriers by increasing efficiency (e.g. by reducing the number of training sessions and exercise tests). In other medical disciplines, the use of algorithms to standardise care and thereby to prevent medical errors and unnecessary costs is already widely accepted [37]. A well-implemented example is the surgical safety checklist, which has shown to improve multiple patient outcomes [38]. In the Netherlands, large-scale implementation of a clinical algorithm for the assessment of patient needs in multidisciplinary CR led to a substantial increase in guideline adherence and a reduction in practice variation [35].

As outlined, the presented algorithms are designed to increase implementation of exercise-based CR programs in clinical practice. However, implementation of these algorithms may still be hampered by the fact that they are not integrated in the ICT systems used in CR centres. Therefore, an additional strategy could be to use the algorithms for the development of a computerised decision support system (CDSS). A CDSS could guide users through the algorithms, helping them with the formation of a personalised, tailored exercise-based CR program. In several trials it was shown that CDSSs improve decisions of individual professionals at, for instance, screening for cancer and management of diabetes [39–42]. Furthermore, CDSSs have also proved to be able to improve guideline adherence [43]. As such, Goud et al. [35] showed that a CDSS based on clinical algorithms improved guideline implementation for CR needs assessment, specifically if the key barrier was the knowledge of professionals. Currently, a CDSS based on a revised set of these algorithms is already integrated in the electronic patient files of several Dutch CR centres. In the future this could facilitate the use of the clinical algorithms for exercise-based CR to be integrated as a CDSS in ICT systems at Dutch CR centres. As such, a trial in ten Dutch CR centres is currently running in which the effect of a CDSS, based on these clinical algorithms, is tested. On the longer term, individual tailoring by clinical algorithms whether or not used in a CDSS could facilitate guideline implementation in practice and improve cost-effectiveness of exercise-based CR programs.

Several limitations should be acknowledged. First, recommendations for the evaluation instrument for the goals ‘reducing exercise-related anxiety’ and ‘developing a physical active lifestyle’, and home-based training during second phase for three exercise-based CR goals were based on expert opinion. Nevertheless, these evaluation instruments have been previously validated and are therefore expected to provide useful information on the progression with respect to individual rehabilitation goals. Secondly, clinical algorithms may not overcome certain external barriers that are not related to awareness or complexity of guidelines. For instance, Bradley et al. reported that poor implementation of recommendations for exercise-based CR programs in Northern Ireland was caused at least partly by a lack of facilities, implicating that also other strategies for better guideline implementation are needed [44]. However, as the algorithms offer alternatives for institutions that lack facilities for symptom-limited exercise testing with gas analysis, we do not believe that these barriers hamper its implementation. Furthermore, it should be noted that the proposed algorithms offer recommendations for exercise-based CR only and that other modalities of CR and secondary prevention, such as psychological treatment, dietary advice and smoking cessation, should also be addressed on an individual basis. Also, the proposed algorithms do not provide advice on maintenance programs after the initial CR phase, typically including behavioural techniques and focusing on incorporating lifestyle changes into daily life, in order to improve long-term adherence to lifestyle modifications [45]. Finally, the algorithms provide no recommendations for patients with heart failure with preserved ejection fraction. Although recent studies focusing on exercise training for these patients showed promising results, recent guidelines do not provide recommendations for this patient category yet [46].

This study presents evidence-based clinical algorithms for exercise-based CR, enabling healthcare professionals in CR to prescribe and evaluate personalised exercise-based CR programs for CAD and CHF patients, based on their individual rehabilitation goals and physical fitness levels. Implementation of these algorithms may result in a reduction of practice variation and improved guideline adherence.
